# The Role of Human Epidermal Growth Factor Receptor 2 (HER2)-Targeted Therapies in Early-Stage Breast Cancer: Current Practices, Treatment De-escalation, and Future Prospects

**DOI:** 10.7759/cureus.55230

**Published:** 2024-02-29

**Authors:** Mohamed Hashem, Shazza Rehman, Mohamed Salhab

**Affiliations:** 1 Breast Surgery, Mid Yorkshire NHS Teaching Trust, Wakefield, GBR; 2 Oncology, Airedale NHS Foundation Trust, Airedale, GBR; 3 Breast Surgery, Bradford Teaching Hospitals NHS Foundation Trust, Bradford, GBR

**Keywords:** pathological complete response (pcr), targeted axillary dissection, breast-conserving surgery, neoadjuvant, axillary management, de-escalation strategies, targeted therapy, her2 positive breast cancer

## Abstract

Human epidermal growth factor receptor 2 (HER2)-targeted therapy has transformed the treatment paradigm for early-stage HER2-positive breast cancer, providing personalized and effective interventions. This comprehensive review delves into the current state of HER2-targeted therapies, emphasizing pivotal clinical trials that have demonstrated their substantial impact on long-term outcomes. Combination therapies that integrate HER2-targeted agents with chemotherapy exhibit enhanced tumor responses, particularly in neoadjuvant settings. Neoadjuvant chemotherapy (NACT) is explored for its role in tumor downsizing, facilitating breast-conserving surgery (BCS), and incorporating oncoplastic solutions to address both oncologic efficacy and aesthetic outcomes. Innovative axillary management post-NACT, such as targeted axillary dissection (TAD), is discussed for minimizing morbidity.

The review further explores the delicate balance between maximal therapy and de-escalation, reflecting recent trends in treatment approaches. The therapeutic landscape of HER2-low breast cancer is examined, highlighting considerations in HER2-positive breast cancer with BReast CAncer gene (BRCA) mutations. Emerging immunotherapeutic strategies, encompassing immune checkpoint inhibitors and chimeric antigen receptor (CAR) T-cell therapy, are discussed in the context of their potential integration into treatment paradigms.

In conclusion, the evolving landscape of HER2-positive early-stage breast cancer treatment, characterized by targeted therapies and multidisciplinary approaches, underscores the need for ongoing research and collaborative efforts. The aim is to refine treatment strategies and enhance patient outcomes in this dynamic and rapidly evolving field.

## Introduction and background

Recent years have seen a surge in interest in human epidermal growth factor receptor (HER) targeted therapy due to its profound effects on the management of several malignancies, most notably breast cancer. The role of HER receptors in the initiation and spread of cancer has become more fully understood. Cancers of the breast, lung, and ovary have all been associated with mutations in or overexpression of HER receptors, particularly HER2. Treatments for HER2-positive breast cancer that target HER2, such as monoclonal antibodies like trastuzumab [1] and pertuzumab [2], have demonstrated impressive efficacy. The concept of personalized medicine, in which treatment choices are made based on a patient's unique genetic profile and tumor features, is best exemplified by breast cancer targeted therapy. Targeted therapy for HER2-positive breast cancer is still evolving, leading to new medications and treatment approaches. The possibility of combining HER2-targeted agents with other forms of treatment, like immunotherapy, radiation therapy, and chemotherapy, is being investigated by researchers. These combination strategies aim to address resistance mechanisms that may arise from single-agent therapy while also improving treatment efficacy.

This article provides a thorough overview of the treatment of HER2-positive early-stage breast cancer to highlight knowledge gaps, illuminate the current state of the most effective treatment approaches, and provide insights into the promising findings of recent research that are more likely to shape future treatment strategies.

Methodology

We conducted a search within PubMed for literature about HER2-positive breast cancer, with a focus on early-stage breast cancer. Priority was given to randomized controlled trials, meta-analyses, and systematic reviews. Guidelines from major professional societies were also reviewed to establish current practice.

## Review

Current HER2-targeted therapies in early-stage breast cancer

Current HER2-targeted therapies in early-stage breast cancer Several clinical trials have shown that HER2-targeted agents have significantly impacted the long-term outcomes and disease-free survival of patients with HER2-positive early-stage breast cancer. These trials have provided essential evidence to guide treatment decisions and improve patient outcomes. Table 1 summarizes some key clinical trials that have contributed to our understanding of the efficacy and safety of HER2-targeted therapies [3-8].

**Table 1:** Summary of landmark HER2 trials. FEC: 5-fluorouracil, epirubicin and cyclophosphamide; T: Docetaxel; H: Herceptin (Trastuzumab); P: Pertuzumab; pCR: Pathological complete response.

Name of the Trial	Year First Published	Study Type	Number of Patients	Study Design	Primary Endpoint	Secondary Endpoint	Neo-adjuvant/Adjuvant Setting	Pathological Complete Response Rate (pCR)	Duration of Follow-Up	Disease-Free Survival	Hazard Ratio	Overall Survival After
HERA Trial [3]	2005	International, multicenter, open-label, phase 3 randomized trial	1694 in the treatment group and 1693 in the control group	1694 women assigned to one year of trastuzumab, and 1693 women assigned to observation. **Both arms had their chemotherapy before randomization.	Disease-free survival	Cardiac safety, overall survival, site of first disease-free–survival event, and time to distant recurrence	Adjuvant: 89%. Neo-Adjuvant: 5%. Combined: 6%.	Not reported	1 year (range, 0 to 36 months)	Trastuzumab: 92.5% Control group: 87%	0.54 (95% CI, 0.43 to 0.67; P<0.0001)	Trastuzumab: 98.3% Control group: 97.8%
NSABP B-31 and NCCTG-N9831 Trials [4]	2005	Randomized control trial	1833 in the treatment group and 1843 in the control group	Treatment group: 4 cycles of doxorubicin and cyclophosphamide followed by paclitaxel and trastuzumab. Control group: 4 cycles of doxorubicin and cyclophosphamide followed by paclitaxel only.	Disease-free survival	Overall survival, time to distant recurrence, death from breast cancer, contralateral breast cancer, and other second primary cancers	Adjuvant setting	None applicable	2 years	Trastuzumab: 87.1% Control group: 75.4%	0.48 (95% CI, 0.39 to 0.59; P<0.0001)	Trastuzumab: 94.3% Control group: 91.7%
NeoSphere Trial [5,6]	2012 (5-year follow-up published in 2016)	International, multicenter, open-label, phase 2 randomized trial	417 eligible patients	Group A: H + T. Group B: P + H + T. Group C: P + H. Group D: P + T.	Pathological complete response	5-year progression-free survival (analyzed in the intention-to-treat population) and disease-free survival	Neo-Adjuvant setting	Group A: 29%. Group B: 45.8%. Group C: 16.8%. Group D: 24%.	5 years	81% (95% CI 72-88) for group A, 84% (72-91) for group B, 80% (70-86) for group C, and 75% (64-83) for group D	Hazard ratios: Group B vs group A: 0.69 [95% CI 0.34-1.40], Group C vs group A: 1.25 [0.68-2.30], Group D vs group B: 2.05 [1.07-3.93]	Not reported
TRYPHAENA Trial [7]	2013	A randomized phase II cardiac safety study	Group A: 73. Group B: 75. Group C: 77.	Group A: FEC + H + P ×3 followed by T + H + P ×3. Group B: FEC ×3 followed by T + H + P ×3. Group C: T + carboplatin + H + P ×6.	Incidence of symptomatic left ventricular systolic dysfunction (LVSD)	Rate of pCR, clinical response rate, time to clinical response, rate of breast-conserving surgery for patients for whom mastectomy was planned before treatment (T2-3), disease-free survival, progression-free survival, and overall survival.	Neo-Adjuvant setting.	Group A: 61.6%. Group B: 57.3%. Group C: 66.2%.	20-21 months	Not reported	Not reported	Not reported
APHINITY Trial [8]	2017 (6-year follow-up in 2021)	Randomized, multicenter, multinational, double-blind, placebo-controlled trial	Treatment group: 2400. Control group: 2405.	Treatment group: Chemotherapy + H + P. Control group: Chemotherapy + H + Placebo. **Duration of anti-HER2 therapy: 1 year.	Invasive-disease–free survival	Overall survival, disease-free survival, relapse-free interval and distant-relapse–free interval, safety, and health-related quality of life	Adjuvant setting.	Not applicable	74 months median follow-up	Treatment group: 91% Control group: 88%	0.76 (95% CI, 0.64 to 0.91)	Treatment group: 95%. Control group: 94%

The first HER2-targeted medication licensed for treating HER2-positive breast cancer was trastuzumab, also known as Herceptin. It is a monoclonal antibody that specifically targets the HER2 receptor, which is amplified or overexpressed in HER2-positive breast cancer. By binding to HER2's extracellular domain, trastuzumab stops downstream signaling [3]. It prevents angiogenesis, cellular survival, and proliferation mediated by HER2. Additionally, trastuzumab stimulates antibody-dependent cellular cytotoxicity (ADCC), triggering the immune system to eliminate HER2-positive cancer cells [9].

Another monoclonal antibody targeting the HER2 receptor is pertuzumab (Perjeta). Unlike trastuzumab, it selectively binds to an alternative domain of the HER2 protein, resulting in augmented and complementary inhibition of HER2. By impeding the dimerization of HER2 with other HER family receptors, predominantly HER3, pertuzumab obstructs subsequent signaling pathways. Through the inhibition of HER2-HER3 heterodimerization, pertuzumab surpasses the level of HER2 inhibition attained by trastuzumab alone.

More recently, Ado-trastuzumab emtansine, commonly known as T-DM1, is being increasingly used in practice. It is an antibody-drug conjugate (ADC) that combines trastuzumab with a cytotoxic agent, Emtansine (DM1). Trastuzumab serves as the targeting moiety, delivering the DM1 payload directly to HER2-positive cancer cells. Emtansine is a potent microtubule-disrupting agent internalized and released within the cancer cells, leading to cell cycle arrest and apoptosis [10].

Combination therapy

Combination therapies involving chemotherapy and HER2-targeted therapy have established themselves as effective treatment modalities for early-stage HER2-positive breast cancer. In the neoadjuvant setting, the NeoSphere and TRYPHAENA trials showed higher rates of pathological complete response (pCR) (45% and up to 66%, respectively), when both were included in combination with chemotherapy, indicating enhanced tumor response [6,7]. Neoadjuvant combination therapy using chemotherapy, trastuzumab, and pertuzumab has become increasingly popular and is recommended in most major professional guidelines.

For patients with HER2-positive breast cancer, positive lymph nodes (cN1), or a tumor size of 2cm or more, the Association of Breast Surgeons in the UK (ABS) suggests administering adriamycin and cyclophosphamide (AC) or epirubicin and cyclophosphamide (EC) in a dose-dense (3 weeks) fashion, followed by paclitaxel (or docetaxel) plus trastuzumab plus pertuzumab as neo-adjuvant chemotherapy (NACT). This is also considered for T1b/T1c N0 if necessary to facilitate breast-conserving surgery (BCS) or permit germline testing [11]. Similarly, for cT > 2cm or positive LNs, the National Cancer Institute in the United States advises the combination of chemotherapy with Trastuzumab and Pertuzumab [12]. Furthermore, for malignancies classified as T1b or higher or N1, the European Society of Medical Oncology (ESMO) advises the concurrent use of dual anti-HER2 therapy and chemotherapy as NACT [13].

In the adjuvant setting, the APHINITY trial demonstrated that the combination of pertuzumab and trastuzumab showed a small but statistically significant improvement in invasive disease-free survival, supporting the use of dual anti-HER2 therapy in the adjuvant setting [8]. In current practice, for patients who achieved pCR and were node-negative, monotherapy with trastuzumab for 14 cycles should suffice. Dual therapy with trastuzumab and pertuzumab is recommended if the patient achieved pCR but was either node-positive pre-NACT or histology showed lymph nodes with scarring indicating a response to treatment [11-13]. For those with residual disease after NACT, the recommendation is to switch therapy to T-DM1, as the KATHERINE trial showed that T-DM1 improves recurrence-free survival (RFS) by 50% when compared with trastuzumab [14].

Tyrosine kinase inhibitor

Other agents are being investigated in the treatment of HER2-positive early breast cancer, such as lapatinib and neratinib. Epidermal growth factor receptor and HER2 receptor are both inhibited by the small molecule kinase inhibitor lapatinib. The available evidence does not support the preoperative administration of lapatinib. The phase III trial CALGB 40601 (NCT00770809) did not show any statistically significant benefit in terms of pCR when lapatinib is added to trastuzumab in combination with Taxane-based chemotherapy [15]. Recent meta-analyses of randomized clinical trials showed that the combination of lapatinib and trastuzumab, in addition to chemotherapy, has survival benefits in terms of RFS and OS [16]. However, no clinical trials have directly compared trastuzumab and pertuzumab versus trastuzumab and lapatinib in combination with chemotherapy.

Neratinib, an irreversible tyrosine kinase iInhibitor in oral form, can be used in the adjuvant setting after one year of adjuvant monotherapy with trastuzumab in patients at a high risk of recurrence. The ExteNET trial, a multicenter, randomized, double-blind, placebo-controlled, phase 3 trial, showed that the use of neratinib after one year of adjuvant trastuzumab improves disease-free sSurvival (DFS) by about 33% after two years of follow-up when compared to placebo (HR: 0.67, CI: 0.50-0.91). This finding is further augmented in ER-positive cancer (HR: 0.51, CI: 0.33-0.77) [17]. There is no current data to support using neratinib in the adjuvant setting if dual anti-HER2 therapy is used pre-operatively.

The role of NACT in de-escalation of breast surgery

A significant advantage of NACT is tumor downsizing or pathological response (pCR), providing substantial evidence to support breast-conserving surgery (BCS) in patients who otherwise would be offered mastectomy [18]. Yamaguchi T et al. demonstrated in a phase II randomized controlled trial that the use of NACT increases the incidence of BCS in HER2 breast cancer by 25.8% [19]. After NACT downstaging, the utilization of BCS did not increase the local recurrence rate [20]. Therefore, NACT has transformed the landscape of breast cancer treatment, providing a platform for innovative surgical approaches that prioritize both oncologic efficacy and aesthetic outcomes.

Mammoplasty, a reconstructive technique commonly used in breast cancer surgery post-NACT, has gained prominence for its ability to achieve symmetrical and aesthetically pleasing results. The downsizing effect of NACT facilitates BCS in cases where mastectomy might have initially been recommended. Mammoplasty allows for the reshaping of the breast while preserving vital oncologic principles. Notably, the utilization of oncoplastic techniques in conjunction with NACT has been associated with improved patient satisfaction and minimized psychosocial impact. By seamlessly integrating oncologic principles with plastic surgery expertise, mammoplasty emerges as a pivotal tool in the surgeon's arsenal, offering a holistic approach to breast cancer surgery that addresses both the disease and its impact on the patients' quality of life. Di Leone A et al. conducted a retrospective study of 297 patients who had NACT before surgery (87 had level 2 mammoplasty oncoplastic surgery level II techniques (OPSII), while 210 had a mastectomy with immediate reconstruction (MIBR)), with a median follow-up of 39.5 months. There was no significant statistical difference between both groups in terms of local, regional, or distant recurrence, as well as overall survival. Moreover, no difference was found in breast satisfaction, although physical well-being was much higher in patients who had OPSII [21]. Similar findings were reported by van la Parra RF et al. regarding the oncological safety of BCS (both simple BCS and mammoplasties) compared to mastectomy after NACT [22].

Chest wall perforator flaps (CWPF) represent another noteworthy advancement in breast cancer surgery post-NACT, especially in cases where extensive tissue resection is required. These flaps, utilizing autologous tissue from the chest wall, offer a versatile solution for breast reconstruction. The “PartBreCon” study, a multicenter retrospective cohort study, that assessed the postoperative and oncological outcomes after different types of CWPF in partial breast reconstruction. The 30-day postoperative complication rate was 12%, and the re-excision rate was 15.9%. The rates for local, regional, and distant recurrence were 1%, 0.6%, and 3.2%, respectively. This study demonstrated that the use of CWPF in partial breast reconstruction is a safe alternative to mastectomy [23]. The use of CWPF is particularly relevant in the context of oncoplastic surgery post-NACT, allowing for the replacement of excised breast tissue with well-vascularized, autologous tissue. This approach not only aids in achieving optimal cosmetic outcomes but also provides a durable and natural reconstruction. The adaptability of CWPF allows surgeons to tailor the reconstruction to the unique anatomical features of each patient, ensuring personalized and aesthetically pleasing results. As breast cancer surgery continues to evolve, the integration of mammoplasty and chest wall perforator flaps stands as a testament to the commitment to comprehensive patient care, emphasizing both oncologic efficacy and enhanced quality of life post-NACT. This will have a positive impact on patients’ mental well-being and increase satisfaction with patients’ perceptions of their body image, as shown in a recent meta-analysis [24]. Ng ET et al. have shown similar results as well [25].

The role of NACT in de-escalation of axillary surgery

Numerous studies have demonstrated the ability of NACT to induce significant axillary downstaging rates. Samiei S et al. conducted a systematic review and meta-analysis, which showed that the pCR for positive axillary lymph nodes after NACT was 60% in ER-negative HER2-positive disease, dropping to 48% if it was triple-positive disease [26]. In recent years, efforts have been made to reduce the intensity of axillary treatment for patients with clinically involved axillary lymph nodes and NACT, due to the morbidity associated with axillary node clearance. One concern of sentinel lymph node biopsy (SLNB) after NACT is the high and unacceptable false-negative rate (FNR). Four prospective, multi-institutional clinical trials examined the feasibility of SLNB after NACT in breast cancer patients with involved lymph nodes [27-30]. The FNR is reported up to 14.2% in the SENTINA trial. However, removing three or more SLN was shown to reduce the FNR down to 9.1% and 7.3% in the ACOSOG Z1071 and SENTINA trials, respectively [27,28]. In a meta-analysis, the FNR was seen to be around 4% when 3 or more SLN are removed [27]. Using a dual-agent SLNB localization technique also was seen to reduce the FNR [31,32]. For patients with positive lymph nodes before NACT, the risk of FNR could be further reduced by clipping the positive node before NACT and targeting this lymph node. Combined SNB and targeted removal of clipped nodes can be performed, a technique called targeted axillary dissection (TAD) [33]. A recent systematic review and pooled analysis demonstrated that the FNR was 6.28% after excision of the clipped node only post-NACT. This figure was brought down to 5.18% when TAD was done [34]. Song YX et al. showed in their meta-analysis that the FNR was 5.1 and 6.3 in TLNB and TAD, respectively, which was not statistically significant [35].

Localization methods of impalpable lesions

In order to facilitate both tumor and positive lymph node localization, several localization modalities have been developed. Initially, this was done by wire localization mostly on the day of surgery, the downside of which includes patient dissatisfaction, wire displacement, and logistical problems in facilitating wire insertion on the day of surgery. Therefore, other methods have been developed; Table 2 summarizes different methods of localization. Currently, no data suggest the superiority of any TAD localization method [36].

**Table 2:** Different types of localization of impalpable breast lesions.

Localization Method	Description	Advantages	Disadvantages
Wire Localization	Thin wire inserted into the breast, leading to the target lesion.	Established method	Patient discomfort
		Direct guidance to the lesion	Risk of wire dislodgement during patient transport or surgery
			Difficulty in precise localization
Radioactive Seed Localization (RSL)	Radioactive seed implanted near the lesion, detected by a handheld gamma probe.	Precise localization	Radiation exposure concerns
		Flexibility in scheduling surgery	Regulatory challenges
		Reduced wire-related complications	
Radiofrequency Identification (RFID) Tags	Tiny RFID tags implanted near the lesion, detected by a specialized probe.	Real-time information on lesion location	Limited widespread adoption due to technology and cost considerations
		Reduced patient discomfort	
		Accurate and efficient excision	
Magnetic Resonance Imaging (MRI)-Guided Localization	Marker placed under MRI guidance to identify the target lesion.	High-resolution imaging	Logistical challenges
		Real-time information	Cost considerations
		Useful for lesions primarily detected through MRI	Limited availability of MRI facilities
Magseed Localization	Magnetic marker (Magseed) inserted into the breast, detected by a magnetic probe.	Improved precision	Cost considerations
		Flexibility in scheduling surgery	Availability and institutional preferences may influence adoption
		Reduced patient discomfort	
		No radioactive materials	
		Streamlined surgical workflow	

Axillary management in node-positive breast cancer after neoadjuvant chemotherapy: navigating the therapeutic dilemma

The management of the axilla in patients who remain node-positive after neoadjuvant chemotherapy (NACT) presents a persistent clinical challenge. The current standard of care dictates axillary lymph node dissection (ALND) in these cases; however, a growing body of evidence questions whether radiotherapy alone could provide adequate local control and prevent recurrence. Addressing this dilemma is crucial for optimizing patient outcomes, minimizing unnecessary procedures and associated morbidities. The AXSANA trial, currently in the recruitment phase, aims to provide definitive answers regarding the optimal management strategy for patients with residual nodal disease post-NACT. By comparing outcomes between ALND or radiotherapy alone in this specific cohort, the trial aspires to guide future clinical practice, offering evidence-based insights into the most judicious approach for axillary management in these patients [37].

In addition to the AXSANA trial, the scientific community eagerly awaits results from trials such as NSABP-B51 (NCT01872975) and ATNEC (NCT04109079). These studies, designed to address nuances in axillary management post-NACT, stand poised to contribute valuable data to the ongoing discourse. Their findings may further refine our understanding of the optimal treatment pathway for patients with persistent nodal disease, influencing both national and international guidelines.

Can surgery be avoided in patients who achieve pCR?

Others advocate for even more conservative approaches, such as random biopsy of the tumor bed. Proponents of this approach suggest that random biopsies of the tumor bed, combined with systemic therapy and radiotherapy to the breast, could be sufficient for patients who achieve a pCR after NACT. This approach could potentially reduce the need for extensive surgery and its associated complications, while still providing adequate local control. The NOSTRA trial (NCT04118192), a prospective non-randomized feasibility study, is designed to evaluate the role of random biopsy of the tumor bed in patients with HER2-positive, ER-negative early-stage breast cancer. The primary endpoint of the trial is the FNR of these random biopsies, and the secondary endpoints include OS, locoregional recurrence-free survival (LRS), distant RFS, and the FNR of SNBx.

The results of the NOSTRA trial are expected to provide valuable insights into the potential role of random biopsy of the tumor bed in the treatment of early breast cancer. If the trial demonstrates that this approach is safe and effective, it could represent a significant shift in the surgical management of this disease.

Challenges and limitations

As with any medical treatment, there will be some challenges and limitations. One of these limitations is the side effects associated with the use of trastuzumab. One of the most significant side effects is cardiac complications such as left ventricular systolic dysfunction (LVSD) or cardiac failure. The HERA trial showed that the risk of cardiac adverse events with trastuzumab is around 4.4% [1]. The Affinity trial demonstrated that the addition of pertuzumab to trastuzumab did not increase cardiac toxicity, with the risk of cardiac failure remaining lower than 1% [8]. Cardiac monitoring with echocardiograms or cardiac MRI has become part of routine practice when considering anti-HER2 therapy. Other common side effects include diarrhea, alopecia, neutropenia, and vomiting.

Another challenge is the development of drug resistance. HER2 cancer cells can develop resistance to trastuzumab through either interference with the binding of trastuzumab to the HER2 receptor, increased activation of downstream signaling pathways of HER2, activation of alternative signaling pathways, or the inability to initiate an immune-mediated mechanism for destroying tumor cells [38]. Understanding the mechanisms of resistance is a crucial step towards the discovery of new drugs to overcome this obstacle.

Ongoing research and future prospects

Balancing Maximal Therapy With De-escalation in Breast Cancer Treatment

In order to attain better results in terms of OS, DFS, and pCR, breast cancer treatment has traditionally followed a trajectory of escalating therapy. Contrarily, there has been an increasing focus in recent years on de-escalation, which is a strategic approach to reduce the intensity of treatment for patients with diseases that pose a minimal to extremely minimal risk. De-escalation is predicated on the understanding that aggressive treatment approaches, despite their potential for greater curative efficacy, can result in substantial adverse effects and diminished quality of life. De-escalation strategies strive to achieve a harmonious equilibrium between optimizing treatment effectiveness and reducing avoidable toxicity by customizing interventions according to the unique risk profile of each patient. In the context of early-stage breast cancer, where a significant proportion of patients have low to very low-risk disease, the de-escalation strategy is especially applicable. The hazards associated with aggressive treatment may not be sufficient to justify its benefits for these patients; therefore, a more individualized approach might be more appropriate.

Management of Low-Risk HER2 Disease

For low-risk breast cancer, specifically patients with HER2-positive T1 tumors that are ER-positive but have negative lymph nodes, treatment de-escalation has become a feasible option to consider. One notable approach involves adjusting adjuvant therapy, particularly through customizing the treatment methodology to minimize potential adverse effects while preserving effectiveness. An interesting alternative is the concurrent use of weekly Paclitaxel and trastuzumab as HER2 monotherapy instead of dual anti-HER2 therapy, which includes chemotherapy. The APT trial, a non-randomized open-label phase 2 trial with comprehensive 10-year follow-up, has validated the safety and efficacy of trastuzumab combined with weekly Paclitaxel in managing small, node-negative HER2-positive breast cancer [39]. This approach provides clinicians with a prudent option for individualized treatment strategies in this subset of patients.

Furthermore, the duration of trastuzumab therapy has been a focus of investigation, and the PERSEPHONE Trial, a phase 3 randomized prospective trial involving over 4000 patients, has provided noteworthy insights. In this trial, patients were randomized to receive either a standard 12-month course or a shortened 6-month regimen of trastuzumab in addition to chemotherapy. The trial successfully demonstrated the non-inferiority of the 6-month duration regarding disease-free survival after a 4-year follow-up period, with rates of 89.4% versus 89.8%, respectively. Importantly, the 6-month group experienced fewer severe adverse effects (19% vs. 24%), highlighting the potential for a shorter duration of treatment to be particularly beneficial for patients with low-risk disease or those prone to treatment-related intolerability [40].

Management of ER-Positive, HER2-Positive Disease

In the realm of ER-positive, HER2-positive disease, an evolving strategy involves incorporating endocrine treatment and/or cyclin-dependent kinase (CDK) 4/6 inhibitors in conjunction with anti-HER2 therapy. While promising results have been observed in the metastatic setting, applying this approach in the neoadjuvant setting remains an area of ongoing investigation. This avenue presents an exciting prospect for tailoring treatment strategies to overcome the resistance that may develop against anti-HER2 therapy, thereby offering a new approach for optimizing therapeutic outcomes in this specific patient population [41].

Therapeutic Landscape of HER2-Low Breast Cancer: Emerging Role of Trastuzumab Deruxtecan

A recently identified subtype of breast cancer, HER2-low breast cancer, is distinguished by its reduced HER2 expression. Although HER2-negative breast malignancies are less aggressive than HER2-positive cancers, HER2-low cancers remain comparatively more aggressive. The definition of HER2-low breast cancer, typically characterized as HER2 1+ or HER2 2+ without FISH amplification, is not universally agreed upon; however, these tumors are currently classified as HER2-negative breast cancer. In recent years, the development of targeted therapies for HER2-negative breast cancer has garnered increasing attention. An example of such treatment is trastuzumab deruxtecan, a combination of the chemotherapy drug deruxtecan and the antibody trastuzumab. The DESTINY-Breast04 clinical trial assessed the safety and efficacy of trastuzumab deruxtecan in individuals diagnosed with HER2-low unresectable metastatic breast cancer. Trastuzumab deruxtecan substantially prolonged progression-free survival (PFS) and overall survival (OS) compared to standard chemotherapy, according to the trial. Based on the findings from the DESTINY-Breast04 trial, trastuzumab deruxtecan appears to be a potentially effective novel treatment for HER2-negative breast cancer [42]. Further investigation is required to validate these results and determine the most effective application of trastuzumab deruxtecan in managing HER2-negative breast cancer.

Therapeutic Considerations for HER2-Positive Breast Cancer With BRCA Mutations

HER2-positive breast cancer constitutes less than 10% of cases with BRCA mutations [43]. This combination carries a poor prognosis with a lower OS at five years [44]. While the OlympiA trial demonstrated that the poly ADP ribose polymerase (PARP) inhibitor olaparib significantly improved DFS and OS in BRCA-mutated HER2-negative breast cancer, their role in BRCA-mutated HER2-positive breast cancer remains undefined [45]. The current standard of care for HER2-positive breast cancer involves anti-HER2 therapy as the first-line treatment, with PARP inhibitors reserved for patients with recurrent disease who have previously received anti-HER2 therapy. However, this approach may not be optimal for patients with BRCA mutations, as PARP inhibitors could potentially offer additional benefits in this specific subgroup. Further research is warranted to investigate the efficacy and safety of PARP inhibitors in the upfront setting for BRCA-mutated HER2-positive breast cancer. Clinical trials specifically designed to address this question are crucial to determine the optimal treatment strategy for this patient population.

Emerging Immunotherapeutic Strategies for HER2-Positive Breast Cancer: A Focus on Checkpoint Inhibitors and Vaccines

In the context of cancer treatment, immunotherapy is a modality that stimulates the immune system to combat cancerous cells. There are two main types of immunotherapy that are used to treat HER2-positive breast cancer:

Immune checkpoint inhibitors: These drugs block the activity of immune checkpoint proteins, which help regulate the immune system [46]. By inhibiting these proteins, immunotherapy drugs can aid the immune system in recognizing and attacking cancer cells. Examples include pembrolizumab and atezolizumab, which are programmed death 1 (PD1) and programmed death ligand 1 (PDL1) inhibitors, respectively.

Chimeric antigen receptor (CAR) T-cell therapy: This type of immunotherapy involves engineering a patient's own T cells to recognize and attack cancer cells [47]. There is growing evidence that a combination of CAR T-cell therapy and PD1 inhibitors is an efficient treatment for HER2-positive breast cancer that has relapsed or developed resistance to other therapies [48].

A recent systematic review by Kyriazoglou A et al. included 14 clinical trials exploring the role of different types of immune checkpoint inhibitors in combination with anti-HER2 therapy and chemotherapy. Two of these trials have already completed recruitment and were in the metastatic or recurrent disease stage. The only trial in the neoadjuvant setting was still in the recruitment phase. Nevertheless, the systematic review concluded that the results were promising but not yet conclusive [49]. The IMpassion050 trial, a randomized Phase III trial, explored the benefit of adding atezolizumab to standard chemotherapy, trastuzumab, and pertuzumab in the neoadjuvant setting on the rate of pCR. There was no difference in pCR rates between the atezolizumab and placebo groups [50]. The trial also reported adverse effects related to Atezolizumab, such as fatigue, vomiting, diarrhea, neutropenia, and immune-related side effects such as rash and hepatitis. Clinical trials such as NCT04650451 and NCT04660929 are currently exploring the benefit of CAR T-cell therapy, while clinical trials (NCT04740918 and NCT03125928) are evaluating the efficacy of PD-L1 inhibitors as immune checkpoint inhibitors. These studies suggest that the combination of anti-HER2 therapy and immunotherapy is a promising new approach to the treatment of HER2-positive breast cancer. Further research is needed to confirm these findings and identify the best way to combine these therapies.

Role of Vaccines in the Treatment of HER2-Positive Breast Cancer

Sensitizing the immune system to HER2-positive breast cancer cells is an additional method. This can be achieved through the administration of whole-cell vaccines or fragments of the HER2 molecule. Alternatively, adoptive cell therapy, which employs the adoptive transfer of immune cells engineered to target HER2-positive cancer cells, is another promising approach. An example of immune cells currently under investigation in clinical trials are tumor-infiltrating lymphocytes (TILs) (NCT01922921 and NCT00847171). Although the current focus of these studies is on HER2-positive metastatic breast cancer, the history of cancer treatment indicates that effective therapies often progress from the metastatic to the early stages. Thus, antibody-mediated therapies may ultimately have a substantial impact on the management of HER2-positive breast cancer in its early stages.

## Conclusions

In conclusion, the evolving nature of anti-HER2 treatment has significantly improved the treatment outcomes for HER2-positive early breast cancer. Additionally, it has promoted the concept of de-escalation therapy in the breast and axilla. Further unsolved questions await the findings of ongoing clinical trials, with additional potential therapeutic modalities looming on the horizon.

## References

[1] Cameron D, Piccart-Gebhart MJ, Gelber RD (2017). 11 years' follow-up of trastuzumab after adjuvant chemotherapy in HER2-positive early breast cancer: final analysis of the HERceptin Adjuvant (HERA) trial. Lancet.

[2] Swain SM, Miles D, Kim SB (2020). Pertuzumab, trastuzumab, and docetaxel for HER2-positive metastatic breast cancer (CLEOPATRA): end-of-study results from a double-blind, randomised, placebo-controlled, phase 3 study. Lancet Oncol.

[3] Piccart-Gebhart MJ, Procter M, Leyland-Jones B (2005). Trastuzumab after adjuvant chemotherapy in HER2-positive breast cancer. N Engl J Med.

[4] Romond EH, Perez EA, Bryant J (2005). Trastuzumab plus adjuvant chemotherapy for operable HER2-positive breast cancer. N Engl J Med.

[5] Gianni L, Pienkowski T, Im YH (2016). 5-year analysis of neoadjuvant pertuzumab and trastuzumab in patients with locally advanced, inflammatory, or early-stage HER2-positive breast cancer (NeoSphere): a multicentre, open-label, phase 2 randomised trial. Lancet Oncol.

[6] Gianni L, Pienkowski T, Im YH (2012). Efficacy and safety of neoadjuvant pertuzumab and trastuzumab in women with locally advanced, inflammatory, or early HER2-positive breast cancer (NeoSphere): a randomised multicentre, open-label, phase 2 trial. Lancet Oncol.

[7] Schneeweiss A, Chia S, Hickish T (2013). Pertuzumab plus trastuzumab in combination with standard neoadjuvant anthracycline-containing and anthracycline-free chemotherapy regimens in patients with HER2-positive early breast cancer: a randomized phase II cardiac safety study (TRYPHAENA). Ann Oncol.

[8] Piccart M, Procter M, Fumagalli D (2021). Adjuvant pertuzumab and trastuzumab in early HER2-positive breast cancer in the APHINITY trial: 6 years' follow-up. J Clin Oncol.

[9] Maadi H, Soheilifar MH, Choi WS, Moshtaghian A, Wang Z (2021). Trastuzumab mechanism of action; 20 years of research to unravel a dilemma. Cancers (Basel).

[10] Lewis Phillips GD, Li G, Dugger DL (2008). Targeting HER2-positive breast cancer with trastuzumab-DM1, an antibody-cytotoxic drug conjugate. Cancer Res.

[11] (2023). Association of Breast Pathology, Association of Breast Surgery, British Society of Breast Radiology, and UK Breast Cancer Group. https://associationofbreastsurgery.org.uk/media/515633/neaoadjuvant-chemotherapy-manual-v1.pdf.

[12] (2023). Breast Cancer Treatment (PDQ®)-Health Professional Version. https://www.cancer.gov/types/breast/hp/breast-treatment-pdq.

[13] Cardoso F, Kyriakides S, Ohno S (2019). Early breast cancer: ESMO Clinical Practice Guidelines for diagnosis, treatment and follow-up. Ann Oncol.

[14] von Minckwitz G, Huang CS, Mano MS (2019). Trastuzumab emtansine for residual invasive HER2-positive breast cancer. N Engl J Med.

[15] Carey LA, Berry DA, Cirrincione CT (2016). Molecular heterogeneity and response to neoadjuvant human epidermal growth factor receptor 2 targeting in CALGB 40601, a randomized phase III trial of paclitaxel plus trastuzumab with or without lapatinib. J Clin Oncol.

[16] Guarneri V, Griguolo G, Miglietta F, Conte PF, Dieci MV, Girardi F (2022). Survival after neoadjuvant therapy with trastuzumab-lapatinib and chemotherapy in patients with HER2-positive early breast cancer: a meta-analysis of randomized trials. ESMO Open.

[17] Chan A, Delaloge S, Holmes FA (2016). Neratinib after trastuzumab-based adjuvant therapy in patients with HER2-positive breast cancer (ExteNET): a multicentre, randomised, double-blind, placebo-controlled, phase 3 trial. Lancet Oncol.

[18] Zhou X, Li Y (2016). Local recurrence after breast-conserving surgery and mastectomy following neoadjuvant chemotherapy for locally advanced breast cancer - a meta-analysis. Breast Care (Basel).

[19] Yamaguchi T, Hozumi Y, Sagara Y (2021). The impact of neoadjuvant systemic therapy on breast conservation rates in patients with HER2-positive breast cancer: surgical results from a phase II randomized controlled trial. Surg Oncol.

[20] Mamtani A, Sevilimedu V, Le T, Morrow M, Barrio AV (2022). Is local recurrence higher among patients who downstage to breast conservation after neoadjuvant chemotherapy?. Cancer.

[21] Di Leone A, Franco A, Terribile DA (2022). Level II oncoplastic surgery as an alternative option to mastectomy with immediate breast reconstruction in the neoadjuvant setting: a multidisciplinary single center experience. Cancers (Basel).

[22] van la Parra RF, Clough KB, Thygesen HH, Levy E, Poulet B, Sarfati I, Nos C (2021). Oncological safety of oncoplastic level II mammoplasties after neoadjuvant chemotherapy for large breast cancers: a matched-cohort analysis. Ann Surg Oncol.

[23] Agrawal A, Romics L, Thekkinkattil D (2023). 'PartBreCon' study. A UK multicentre retrospective cohort study to assess outcomes following PARTial BREast reCONstruction with chest wall perforator flaps. Breast.

[24] Panayi AC, Knoedler S, Knoedler L (2024). Patient-reported outcomes utilizing the breast-Q questionnaire after breast conserving surgery with and without oncoplastic breast surgery: a systematic review and meta-analysis. Aesthet Surg J.

[25] Ng ET, Ang RZ, Tran BX (2019). Comparing quality of life in breast cancer patients who underwent mastectomy versus breast-conserving surgery: a meta-analysis. Int J Environ Res Public Health.

[26] Samiei S, Simons JM, Engelen SM, Beets-Tan RG, Classe JM, Smidt ML (2021). Axillary pathologic complete response after neoadjuvant systemic therapy by breast cancer subtype in patients with initially clinically node-positive disease: a systematic review and meta-analysis. JAMA Surg.

[27] Kuehn T, Bauerfeind I, Fehm T (2013). Sentinel-lymph-node biopsy in patients with breast cancer before and after neoadjuvant chemotherapy (SENTINA): a prospective, multicentre cohort study. Lancet Oncol.

[28] Boughey JC, Suman VJ, Mittendorf EA (2013). Sentinel lymph node surgery after neoadjuvant chemotherapy in patients with node-positive breast cancer: the ACOSOG Z1071 (Alliance) clinical trial. JAMA.

[29] Boileau JF, Poirier B, Basik M (2015). Sentinel node biopsy after neoadjuvant chemotherapy in biopsy-proven node-positive breast cancer: the SN FNAC study. J Clin Oncol.

[30] Classe JM, Loaec C, Gimbergues P (2019). Sentinel lymph node biopsy without axillary lymphadenectomy after neoadjuvant chemotherapy is accurate and safe for selected patients: the GANEA 2 study. Breast Cancer Res Treat.

[31] Tee SR, Devane LA, Evoy D, Rothwell J, Geraghty J, Prichard RS, McDermott EW (2018). Meta-analysis of sentinel lymph node biopsy after neoadjuvant chemotherapy in patients with initial biopsy-proven node-positive breast cancer. Br J Surg.

[32] Cao S, Liu X, Cui J (2021). Feasibility and reliability of sentinel lymph node biopsy after neoadjuvant chemotherapy in breast cancer patients with positive axillary nodes at initial diagnosis: an up-to-date meta-analysis of 3,578 patients. Breast.

[33] Caudle AS, Yang WT, Krishnamurthy S (2016). Improved axillary evaluation following neoadjuvant therapy for patients with node-positive breast cancer using selective evaluation of clipped nodes: implementation of targeted axillary dissection. J Clin Oncol.

[34] Swarnkar PK, Tayeh S, Michell MJ, Mokbel K (2021). The evolving role of marked lymph node biopsy (MLNB) and targeted axillary dissection (TAD) after neoadjuvant chemotherapy (NACT) for node-positive breast cancer: systematic review and pooled analysis. Cancers (Basel).

[35] Song YX, Xu Z, Liang MX (2022). Diagnostic accuracy of de-escalated surgical procedure in axilla for node-positive breast cancer patients treated with neoadjuvant systemic therapy: a systematic review and meta-analysis. Cancer Med.

[36] Žatecký J, Coufal O, Zapletal O (2023). Ideal marker for targeted axillary dissection (IMTAD): a prospective multicentre trial. World J Surg Oncol.

[37] Banys-Paluchowski M, Gasparri ML, de Boniface J (2021). Surgical management of the axilla in clinically node-positive breast cancer patients converting to clinical node negativity through neoadjuvant chemotherapy: current status, knowledge gaps, and rationale for the EUBREAST-03 AXSANA study. Cancers (Basel).

[38] Pohlmann PR, Mayer IA, Mernaugh R (2009). Resistance to trastuzumab in breast cancer. Clin Cancer Res.

[39] Tolaney SM, Tarantino P, Graham N (2023). Adjuvant paclitaxel and trastuzumab for node-negative, HER2-positive breast cancer: final 10-year analysis of the open-label, single-arm, phase 2 APT trial. Lancet Oncol.

[40] Earl HM, Hiller L, Vallier AL (2019). 6 versus 12 months of adjuvant trastuzumab for HER2-positive early breast cancer (PERSEPHONE): 4-year disease-free survival results of a randomised phase 3 non-inferiority trial. Lancet.

[41] Koirala N, Dey N, Aske J, De P (2022). Targeting cell cycle progression in HER2+ breast cancer: an emerging treatment opportunity. Int J Mol Sci.

[42] Modi S, Jacot W, Yamashita T (2022). Trastuzumab deruxtecan in previously treated HER2-low advanced breast cancer. N Engl J Med.

[43] Tomasello G, Gambini D, Petrelli F (2022). Characterization of the HER2 status in BRCA-mutated breast cancer: a single institutional series and systematic review with pooled analysis. ESMO Open.

[44] Viansone A, Pellegrino B, Omarini C (2022). Prognostic significance of germline BRCA mutations in patients with HER2-POSITIVE breast cancer. Breast.

[45] Geyer CE Jr, Garber JE, Gelber RD (2022). Overall survival in the OlympiA phase III trial of adjuvant olaparib in patients with germline pathogenic variants in BRCA1/2 and high-risk, early breast cancer. Ann Oncol.

[46] Salmaninejad A, Valilou SF, Shabgah AG, Aslani S, Alimardani M, Pasdar A, Sahebkar A (2019). PD-1/PD-L1 pathway: basic biology and role in cancer immunotherapy. J Cell Physiol.

[47] Riccio G, Ricardo AR, Passariello M (2019). T-cell activating tribodies as a novel approach for efficient killing of ErbB2-positive cancer cells. J Immunother.

[48] Li H, Yuan W, Bin S (2020). Overcome trastuzumab resistance of breast cancer using anti-HER2 chimeric antigen receptor T cells and PD1 blockade. Am J Cancer Res.

[49] Kyriazoglou A, Kaparelou M, Goumas G (2022). Immunotherapy in HER2-positive breast cancer: a systematic review. Breast Care (Basel).

[50] Huober J, Barrios CH, Niikura N (2022). Atezolizumab with neoadjuvant anti-human epidermal growth factor receptor 2 therapy and chemotherapy in human epidermal growth factor receptor 2-positive early breast cancer: primary results of the randomized phase III IMpassion050 trial. J Clin Oncol.

